# The Effect of Antioxidant Supplementation on Operated or Non-Operated Varicocele-Associated Infertility: A Systematic Review and Meta-Analysis

**DOI:** 10.3390/antiox10071067

**Published:** 2021-07-01

**Authors:** Nikolaos Pyrgidis, Ioannis Sokolakis, Vasileios Palapelas, Maksim Tishukov, Ioannis Mykoniatis, Evangelos N. Symeonidis, Athanasios Zachariou, Aris Kaltsas, Nikolaos Sofikitis, Georgios Hatzichristodoulou, Chara Tsiampali, Fotios Dimitriadis

**Affiliations:** 1 Department of Urology, Martha-Maria Hospital Nuremberg, 90491 Nuremberg, Germany; nikospyrgidis@gmail.com (N.P.); sokolakisi@gmail.com (I.S.); drhatzichristodoulou@gmail.com (G.H.); 2 3rd Department of Obstetrics and Gynecology, School of Medicine, Aristotle University, 54124 Thessaloniki, Greece; palapelas@the.forthnet.gr; 3 Urology Department, School of Medicine, Faculty of Health Sciences, Aristotle University of Thessaloniki, 54124 Thessaloniki, Greece; tishukov.maksim@gmail.com (M.T.); g_mikoniatis@hotmail.com (I.M.); evansimeonidis@gmail.com (E.N.S.); 4 Department of Urology, School of Medicine, Ioannina University, 45110 Ioannina, Greece; zahariou@otenet.gr (A.Z.); ares-kaltsas@hotmail.com (A.K.); v.sofikitis@hotmail.com (N.S.); 5 Private Pharmacy, 54250 Thessaloniki, Greece; x.tsiampali@gmail.com

**Keywords:** varicocele, varicocelectomy, infertility, antioxidants, meta-analysis

## Abstract

In patients with varicocele-associated infertility, the effect of antioxidant supplementation on fertility is unknown. We performed a systematic review and meta-analysis to explore their role in patients with operated or non-operated varicocele. We searched major databases and sources of grey literature until May 2021 (PROSPERO: CRD42021248195). We included 14 studies (980 individuals) in the systematic review. Of the 14 studies, 2 explored the effect of antioxidant supplementation in patients with non-operated varicocele, 1 compared antioxidants versus surgical repair of varicocele, while 11 explored antioxidants after surgical repair of varicocele and were also included in the meta-analysis. Regarding pregnancy rates, no significant differences were demonstrated after treatment with antioxidants versus no treatment at three (OR: 2.28, 95% CI: 0.7–7.48) and six months (OR: 1.88, 95% CI: 0.62–5.72). Accordingly, contradictory findings were reported in sperm concentration, morphology, and motility, as well as DNA fragmentation. Our findings indicate that antioxidant supplementation does not improve pregnancy rates and semen parameters in patients with varicocele-associated infertility, in the absence of previous screening for oxidative stress. Based on the previous notion, most included studies also raised methodological concerns. Therefore, definitive conclusions about the efficacy of antioxidant supplementation in this setting cannot be drawn and further research on the field is mandatory.

## 1. Introduction

Varicocele is defined as the abnormal dilation of the pampiniform plexus caused by blood reflux from the testicular vein in patients with congenital insufficient or absent venous valves [1]. Most men with varicocele present normal semen parameters and can father children [2]. Still, varicocele is considered the most common correctable cause of male infertility with an overall prevalence of 25% in men with abnormal semen parameters and 35–40% in infertile men [3]. The exact mechanism of varicocele-associated infertility is multifactorial and not fully understood [4]. It has been postulated that scrotal hyperthermia, hypoxia, reflux of toxic metabolites, and increased intravenous pressure induce oxidative stress and, in turn, lead to testicular dysfunction and infertility [5,6].

Even though surgical repair is considered the treatment of choice in patients with varicocele-associated infertility [7], the central role of oxidative stress in the pathophysiology of varicocele has sparked the debate regarding the efficacy of antioxidant supplementation in this context [8]. Antioxidants are a group of widely available nutraceuticals, such as vitamins, micronutrients, and minerals, that reduce the oxidative insult by scavenging the excess production of free radicals and by disrupting lipid peroxidation or other cascades via a variety of mechanisms [9]. A Cochrane meta-analysis has proposed, with a low level of evidence, that antioxidant supplementation may improve live-birth and pregnancy rates, as well as semen parameters in males with any cause of infertility [10]. However, recently, several relevant and high-quality randomized controlled trials (RCTs) suggested the contrary [11,12,13].

In patients with varicocele-associated infertility, it remains uncaptured whether antioxidants improve semen parameters and pregnancy rates [14,15]. In this context, we performed a systematic review and meta-analysis aiming to explore the role of antioxidants solely in patients with operated or non-operated varicocele and to compare, in this setting, the efficacy and safety of antioxidant supplementation versus operation.

## 2. Materials and Methods

### 2.1. Search Strategy

Our study was performed based on the principles of the Cochrane Handbook for Systematic Reviews of Interventions and the PRISMA statement [16,17]. All materials and methods of this systematic review and meta-analysis were a priori documented in a protocol registered at PROSPERO (ID: CRD42021248195). Two authors (I.S. and M.T.) systematically searched for studies assessing the effect of antioxidants on varicocele-associated infertility published in PubMed, Cochrane Library, Web of Science or Scopus database from inception to May 2021. Accordingly, the two authors hand-searched important sources of grey literature, including clinical trial registries and published abstracts from major conferences on the matter. They also perused the reference lists of all eligible studies and relevant reviews. The applied search strategy is presented in Data Supplement 1.

### 2.2. Eligibility Criteria

Our predefined inclusion criteria comprised RCTs or prospective interventional studies assessing pregnancy rates, sperm parameters, or adverse events after antioxidant treatment versus: (i) placebo or no treatment in patients undergoing surgical repair of varicocele; (ii) placebo or no treatment in patients with non-operated varicocele; and (iii) surgical repair of varicocele in patients with varicocele-associated infertility. On the contrary, we excluded the following: (i) comparative studies evaluating the role of non-antioxidants on varicocele-associated infertility; (ii) studies enrolling men with any cause of infertility who received any other fertility-enhancing drugs, plant extracts, or herbal substances; (iii) retrospective or non-comparative studies; and (iv) animal or molecular studies exploring the role of antioxidants in varicocele-associated infertility. Accordingly, when records with overlapping patient populations were identified, only the most recent study was included.

### 2.3. Data Acquisition and Risk of Bias

Two authors (N.P. and M.T.) independently implemented a three-step screening of the title, abstract, and full-text of all identified studies based on the eligibility criteria. Any disagreements were resolved by consensus. Data concerning study and patient characteristics, type of antioxidant therapy, duration of treatment, as well as outcomes regarding fertility and semen parameters or adverse events of all included records were tabulated in a predefined Microsoft Excel spreadsheet developed in consultation with all co-authors. Moreover, the risk of bias of all included RCTs was evaluated based on the risk of bias (RoB) 2 tool, whereas the risk of bias of all non-RCTs was estimated according to the Robins–I tool [18,19].

### 2.4. Data Synthesis and Statistical Analysis

We produced a qualitative synthesis of the main results extracted from the included studies. More specifically, our primary outcome was the effect of antioxidant supplementation on pregnancy rate in patients undergoing surgical repair of varicocele. Secondary outcomes included the following: (i) the effect of antioxidant supplementation on semen parameters (concentration, motility, morphology, and DNA fragmentation) and safety in patients undergoing surgical repair of varicocele, (ii) the effect of antioxidant supplementation on fertility in patients with non-operated varicocele, and (iii) the effect of antioxidant supplementation versus surgical repair of varicocele in patients with varicocele-associated infertility.

Based on data availability, we undertook an inverse variance, random effects meta-analysis of odds ratios (ORs) to determine the pregnancy rate after antioxidant supplementation versus no treatment in patients undergoing surgical repair of varicocele at three and six months of treatment. Accordingly, we performed an inverse variance, random effects meta-analysis of weighted mean differences (WMDs) in patients undergoing surgical repair of varicocele to determine the effect of antioxidant supplementation versus no treatment or placebo on semen parameters (concentration, motility, morphology, and DNA fragmentation) at three and six months of treatment. For this purpose, we also conducted a subgroup analysis based on the comparator arm (antioxidant supplementation versus placebo or no treatment).

Heterogeneity for all meta-analytic effects was determined based on the I^2^, and its significance was estimated with the *p*-value of the Cochran’s Q test [20]. Due to the small number of included studies, we could not address the potential publication bias [21]. Furthermore, we employed the GRADE system to evaluate the overall strength of evidence for all meta-analytic effects. In particular, two authors (N.P. and I.S.) estimated the risk of bias, inconsistency, indirectness, imprecision, and publication bias of the included studies [22]. We performed the statistical analyses using the R statistical software (version 3.6.3, R Core Team). For all outcomes, 95% confidence intervals (CIs) were reported and *p*-values lower than 0.05 were considered statistically significant.

## 3. Results

### 3.1. Study Selection, Study Characteristics, and Quality Assessment

**Figure 1:**
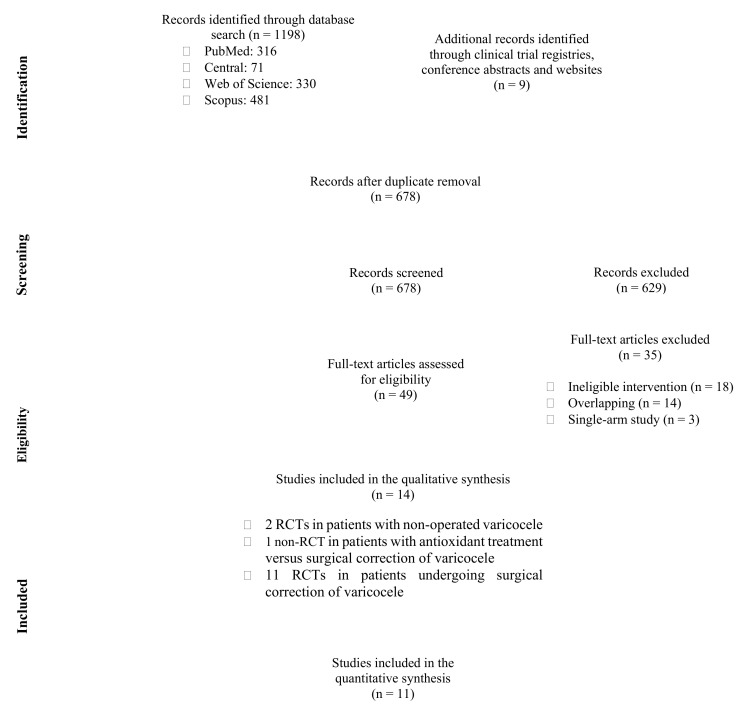
Flow diagram of study selection process. RCT: randomized controlled trial.

Our systematic literature search identified 678 unique studies, yielding 49 eligible articles for full-text evaluation after title and abstract screening. Ultimately, 14 studies (13 RCTs and one non-RCT) were included in the qualitative synthesis of the present systematic review and meta-analysis. In particular, 2 RCTs explored the effect of antioxidant treatment in patients with non-operated varicocele versus placebo [23,24], 1 non-RCT explored the effect of antioxidant treatment versus surgical repair of varicocele [25], while 11 RCTs explored the effect of antioxidant treatment versus placebo or no further postoperative treatment in patients undergoing surgical repair of varicocele and were also included in the meta-analysis [26,27,28,29,30,31,32,33,34,35,36]. No studies were identified addressing the effect of antioxidant treatment versus observation only in patients with non-operated varicocele. The step-by-step study selection process is illustrated in Figure 1 and the reference list of all excluded studies with reasons for exclusion is presented in Data Supplement 2.

A total of 980 individuals with a mean age of 29.8 ± 6 years were included in the systematic review. The presence of left-sided varicocele was confirmed both clinically and sonographically. All studies considered patients with varicocele-associated infertility that were evaluated between 3 and 12 months in the course of treatment with antioxidants. Only the study of Cavallini et al. excluded a female infertility factor by performing the most contemporary relevant assays [24]. Moreover, across all studies, the administered antioxidants displayed high heterogeneity in terms of dosage, intake frequency, and type and number of active substances. None of the included studies measured the direct effect of treatment on oxidative stress. Still, all studies reported mild or no adverse events, and no treatment-related dropouts were observed. The corresponding baseline characteristics of all included studies are depicted in Table 1.

Regarding quality assessment, based on the RoB 2 tool, 2 RCTs were considered at low risk of bias, 1 RCT with some concerns, and 10 RCTs at high risk of bias (Data Supplement 3). Accordingly, based on the Robins–I tool, the included non-RCT was considered at high risk of bias (Data Supplement 4).

### 3.2. Effect of Antioxidant Treatment on Fertility in Patients Undergoing Surgical Repair of Varicocele

**Figure 2:**
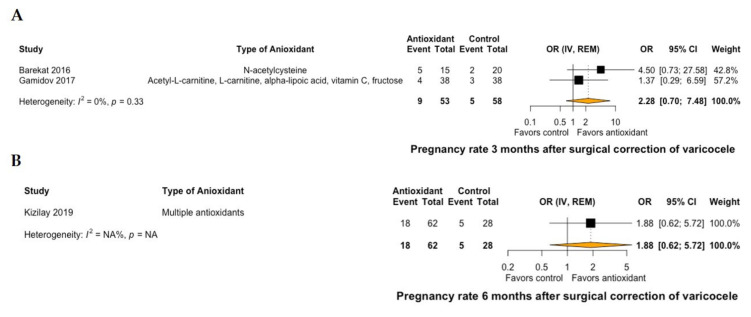
Forest plot of pregnancy rate at 3 months (**A**) and 6 months (**B**) of patients with surgical correction of varicocele and treatment with antioxidants versus no treatment. CI: confidence interval; IV: inverse variance; OR: odds ratio; REM: random effects model.

A total of two RCTs compared the effect of antioxidant treatment versus placebo in patients undergoing surgical repair of varicocele [26,28], whereas nine RCTs compared antioxidants versus no treatment after surgery [27,29,30,31,32,33,34,35,36]. None of the available studies provided outcomes on live-birth rates. Regarding pregnancy rates, two RCTs compared antioxidant treatment versus no postoperative treatment at 3 months [27,31], and one RCT compared antioxidant treatment versus no postoperative treatment at 6 months [32]. No significant differences were demonstrated at both time points after treatment with antioxidants (OR: 2.28, 95% CI: 0.7 to 7.48, I^2^ = 0% at 3 months, Figure 2A; OR: 1.88, 95% CI: 0.62 to 5.72 at 6 months, Figure 2B).

**Figure 3:**
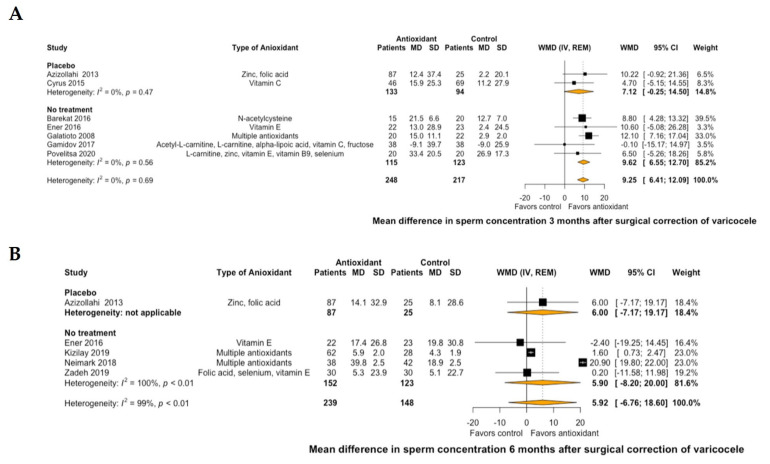
Forest plot of mean difference in sperm concentration at 3 months (**A**) and 6 months (**B**) after treatment with antioxidants versus placebo or no treatment in patients with surgical correction of varicocele. CI: confidence interval; IV: inverse variance; MD: mean difference; REM: random effects model; SD: standard deviation; WMD: weighted mean difference.

**Figure 4:**
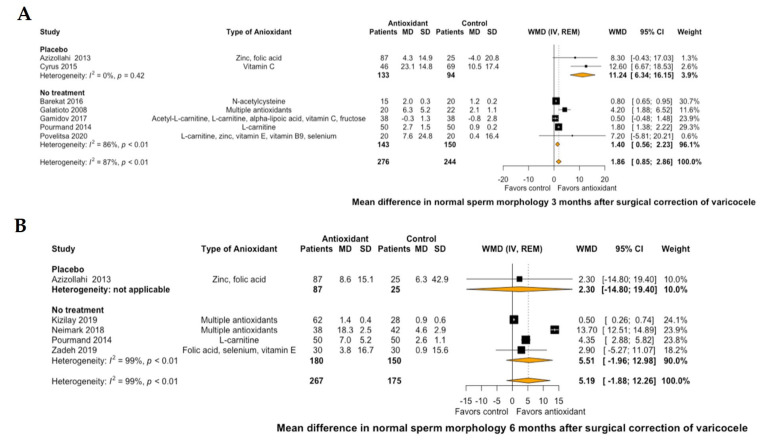
Forest plot of mean difference in normal sperm morphology at 3 months (**A**) and 6 months (**B**) after treatment with antioxidants versus placebo or no treatment in patients with surgical correction of varicocele. CI: confidence interval; IV: inverse variance; MD: mean difference; REM: random effects model; SD: standard deviation; WMD: weighted mean difference.

Regarding semen parameters, antioxidant treatment led to a significant improvement of mean sperm concentration by 9.25 10^6^/mL (95% CI: 6.41 to 12.09, I^2^ = 0%) at 3 months of treatment compared to placebo or no further postoperative treatment (Figure 3A). This significant improvement was not demonstrated at 6 months of antioxidant treatment (mean sperm concentration: 5.92 10^6^/mL, 95% CI: −6.76 to 18.6, I^2^ = 99%, Figure 3B). Similarly, antioxidant treatment led to a significant improvement of mean normal sperm morphology by 1.86% (95% CI: 0.85 to 2.86, I^2^ = 87%) at 3 months of treatment compared to placebo or no further postoperative treatment (Figure 4A). However, this significant improvement was not also demonstrated at 6 months of antioxidant treatment (mean normal sperm morphology: 5.19%, 95% CI: -1.88 to 12.26, I^2^ = 99%, Figure 4B).

At three months of treatment with antioxidants, the total sperm motility improved by a mean of 7.33% (95% CI: 3.27 to 11.38, I^2^ = 83%,  Data Supplement 5) and the progressive sperm motility by 0.01% (95% CI: −4.92 to 4.94, I^2^ = 10%, Data Supplement 6) compared to placebo or no further postoperative treatment. Accordingly, at six months, the total sperm motility improved by a mean of 4.61% (95% CI: −5.1 to 14.32, I^2^ = 98%, Data Supplement 5) and the progressive sperm motility by 2.97% (95% CI: 0.58 to 5.35, I^2^ = 0%, Data Supplement 6) compared to placebo or no further postoperative treatment. Moreover, compared to no further postoperative treatment, antioxidant supplementation reduced DNA fragmentation by 3.07% (95% CI: 0.76 to 5.38, I^2^ = 92%, Data Supplement 7) at three months and by 7.5% (95% CI: −5.24 to 20.24, I^2^ = 99%, Data Supplement 7) at six months.

### 3.3. Effect of Antioxidant Treatment on Fertility in Patients with Non-Operated Varicocele

A total of two RCTs evaluated the efficacy of antioxidant treatment versus placebo in patients with non-operated varicocele leading to infertility [23,24]. Both studies reported negligible adverse events that did not result to any treatment-related dropouts. Busetto et al. compared pregnancy rates and semen parameters after a combination of multiple antioxidants in 21 patients versus placebo in 24 patients. At six months in the course of therapy, a significant difference in favor of antioxidant treatment was only demonstrated in the total sperm motility and not in pregnancy rates [23]. Accordingly, Cavallini et al. compared a combination of 2 g L-carnitine and 0.5 g acetyl-L-carnitine in 62 patients versus placebo in 71 patients. At both 3 and 6 months in the course of therapy, sperm concentration, morphology, and motility significantly improved after antioxidant treatment. However, in a subgroup analysis of patients with severe varicocele, these positive findings of antioxidant treatment could not be demonstrated [24].

Despite the low methodological quality, the rather small follow-up, the contradictory findings, and the differences in dosage and type of administered antioxidant in both studies, the authors concluded that antioxidant supplementation could be effective when implemented in strategies aiming to enhance fertility. Still, it should be highlighted that the apparent limitations of both studies restricted the extrapolation of their findings and do not support the conclusions of the authors. Therefore, both studies may only serve as a valuable motive for the design and implementation of further high-quality studies or, ideally, multicenter, double-blind RCTs.

### 3.4. Antioxidant Treatment Versus Surgical Repair in Patients with Varicocele

To date, only one non-RCT has explored the efficacy of antioxidant treatment versus surgical repair of varicocele in patients with varicocele-associated infertility [25]. More specifically, the authors recruited 62 patients with grade II or higher varicocele that either orally received 250 mg L-carnitine four times a day for six months or underwent inguinal varicocelectomy. At six months, no significant differences were demonstrated in terms of sperm count, concentration, morphology, and motility between patients undergoing conservative versus surgical treatment. The authors concluded that oral L-carnitine may be as effective as varicocelectomy in improving semen parameters in patients with grade II or higher varicocele and, therefore, it can be used as an alternative to surgery.

Still, the findings of the study by Sofimajidpour et al. were tempered by multiple limitations. In particular, the authors performed a single-center study with a relatively short follow-up that assessed semen parameters only at one time point. Furthermore, they did not compare the pregnancy rates between the two groups and did not evaluate the safety of the two treatment modalities. Of note, given that the authors did not randomize participants, the two groups displayed significant differences in terms of baseline characteristics such as varicocele severity or pre-treatment semen parameters [25].

### 3.5. Grading of Evidence

Even though the significance of all outcomes was deemed important, the certainty of provided evidence was considered low or very low. More specifically, the high risk of bias of most included RCTs, the small number of included studies, the restricted sample size, as well as the high heterogeneity of some outcomes downgraded the overall strength of evidence. The detailed grading of evidence for all outcomes is summarized in Data Supplement 8.

## 4. Discussion

The findings of the present systematic review and meta-analysis suggest that, based on the available literature, antioxidant supplementation does not seem to improve pregnancy rate, semen parameters, or DNA integrity in patients with varicocele-associated infertility. More specifically, in patients with surgically corrected varicocele, our analyses demonstrated no significant differences in pregnancy rates at three and six months of treatment with antioxidants. Accordingly, in this patient population, contradictory findings were reported in sperm concentration, morphology, and motility, as well as DNA fragmentation. Additionally, the scarce body of literature is also inconclusive about the role of antioxidants in patients with non-operated varicocele. Of note, antioxidants are a safe treatment modality and, therefore, their role compared to surgical treatment in patients with varicocele-associated infertility should be addressed in future studies. Still, it should be stressed that antioxidant supplementation was given to patients in the absence of any evidence that they were actually suffering from oxidative stress. As a result, definitive conclusions about the efficacy of the treatment cannot be drawn.

It should be highlighted that, in males with varicocele-associated infertility, live-birth and pregnancy rates are considered the most reliable and robust parameters when exploring the efficacy of a treatment on male fertility [37], though the female fertility potential represents the other arm of the couple’s fertility. Given that none of the identified trials assessed live-birth rates and that only three studies reported pregnancy rates, further RCTs are mandatory to establish the role of antioxidant supplementation in this setting. However, data from high-quality, high-volume, or multicenter RCTs recruiting couples with any cause of male factor infertility suggest that antioxidants in the form of monotherapy or combination therapy do not improve in vivo pregnancy or live-birth rates when prescribed to patients without previous assessment of the oxidative stress [11,12,13].

Semen analysis is only a surrogate parameter of fertility, as it cannot precisely distinguish fertile from infertile men [38]. Based on the previous notion, the standard parameters of the semen analysis cannot predict the sperm fertilizing ability both in vitro and in vivo [38]. Even though lower values of each semen parameter increase the likelihood that it may contribute to male infertility [39], semen analysis varies over time and is influenced by multiple factors such as duration of ejaculation abstinence, testicular volume, and paternal age and characteristics [40]. Given that, and based on our analyses, sperm concentration, morphology, and motility, as well as DNA fragmentation were improved only for some time points in patients with surgically corrected varicocele; thus, definite conclusions about the role of antioxidants in this setting cannot be drawn. Still, the authors of the included studies did not screen participants for oxidative stress and, thus, the antioxidant therapy was unjustified. Therefore, studies measuring the direct effect of treatment on oxidative stress and on sperm functional assays (i.e., hypoosmotic swelling test, hemi-zona assay, zona-free hamster oocyte sperm penetration assay) are necessary [41,42].

Additionally, in patients with non-operated varicocele, the available evidence does not support the use of antioxidants in the absence of previous screening for oxidative stress. Compared to placebo, antioxidants improved semen parameters but did not attain higher pregnancy rates. Similarly, in the only available comparative study between antioxidants and surgery, the authors did not evaluate live-birth or pregnancy rates. Still, given that varicocele repair has been criticized for limited efficacy and increased complications when the correct indication is not established [43], the design and implementation of high-quality, long-term comparative studies selecting patients on the basis of an oxidative stress marker and comparing antioxidants versus surgery may showcase antioxidant supplementation as a safe and effective alternative to surgery.

There is currently discordance and, hence, variation in the clinical management of varicocele. Guideline recommendations suggest that it should only be corrected in men with clinical varicocele, abnormal semen parameters, and otherwise unexplained infertility in a couple where the female partner displays good ovarian reserve to attain fertility [44]. On the contrary, any other surgical repair of varicocele harbors the risk of overtreatment and should be avoided [45]. Within this framework, our findings suggest that antioxidants are safe and may be implemented as a surrogate before opting for surgical treatment, whereas in patients that have already undergone surgery, their efficacy is limited without previous assessment of the oxidative stress [46].

In the absence of well-designed observational or randomized studies assessing patients for oxidative stress, we provide the first systematic review and meta-analysis focusing solely on patients with varicocele-associated infertility and highlighting the current gaps in the literature. Ideally, a high-quality study focusing on a well-characterized group of patients presenting with left-sided varicocele and evidence of oxidative stress in the spermatozoa is mandatory to corroborate our findings. This study should divide participants into four groups: (i) no treatment, (ii) surgical correction of varicocele, (iii) treatment with antioxidants, and (iv) treatment with antioxidants and surgery. All patients should then be evaluated in terms of: (i) live birth rate, (ii) measures of oxidative stress in the spermatozoa, and (iii) semen quality.

However, it should be stressed that the findings of the present systematic review and meta-analysis are mitigated by multiple limitations. First of all, the included studies displayed significant heterogeneity in terms of antioxidant supplementation. In particular, all included studies administered different combinations of antioxidants and, therefore, a subgroup analysis based on types of antioxidant supplementation could not be performed. Of interest, most included studies raised methodological concerns. This problem predominantly stemmed from the small number of included participants, the poor methods of reporting randomization, the relatively short follow-up, the high attrition rates, the restricted number of events, and the absence of hard outcomes such as live-birth rates. It should be noted that there were significant differences in the pre-treatment values of semen parameters between the two groups, which may allow us to hypothesize that there were also significant differences in Leydig and Sertoli cellular secretory function before treatment between the two groups. Therefore, comparisons of the endocrine or exocrine responses to different pharmaceutical or surgical treatments of testes may not be valid. Accordingly, some important parameters such as grade of varicocele, fertility of partner, applied technique for varicocele repair, and efficacy of antioxidants on oxidative stress remained unreported in the included studies. Furthermore, the clinical significance of our findings is limited, because none of the included studies evaluated the effect of antioxidants on sperm functional assays, which display a stronger correlation to sperm reproductive capacity compared to semen parameters.

## 5. Conclusions

Our findings indicate that antioxidant supplementation does not improve pregnancy rates and semen parameters in patients with surgically corrected varicocele in the absence of previous oxidative stress screening. Accordingly, in patients with non-operated varicocele, antioxidant supplementation may be a promising treatment modality, but the available literature on the field is scarce, because no high-quality clinical trials have been conducted yet. Overall, the level of evidence for all evaluated outcomes was also deemed low or very low due to the methodological concerns raised by most of the included studies. Therefore, no recommendations can be implemented regarding the optimal type of antioxidant, its dosage and duration of treatment. Unless large, high-quality, long-term, head-to-head RCTs are conducted in patients with documented oxidative stress, the administration of antioxidants will mostly remain empirical.

## Figures and Tables

**Table 1:** Baseline characteristics of the included studies.

First Author, Year of Publication	Type of Study	Population	Type of Antioxidant Supplementation	Duration of Treatment	Participants (*n*)	Adverse Events
Azizollahi 2013 [26]	RCT	Patients with surgically corrected clinical grade III varicocele and infertility receiving antioxidants × 1/day vs. placebo	66 mg zinc 5 mg folic acid 66 mg zinc and 5mg folic acid	6 months	32 vs. 25 26 vs. 25 29 vs. 25	No AEs or AE-related dropouts were reported in both groups
Barekat 2016 [27]	RCT	Patients with surgically corrected clinical grade II or III varicocele and infertility receiving antioxidants × 1/day vs. no treatment	200 mg N-acetylcysteine	3 months	15 vs. 20	No AEs or AE-related dropouts were reported in both groups
Busetto 2018 [23]	RCT	Patients with non-operated clinical grade I–III varicocele and infertility receiving antioxidants × 2/day vs. placebo	1 g L-carnitine, 725 mg fumarate, 0.5 g acetyl-L-carnitine, 1 g fructose, 20 mg CoQ10, 90 mg vitamin C, 10 mg zinc, 200 μg folic acid and 1.5 μg vitamin B12	6 months	21 vs. 24	Nausea, vertigo, or headache in patients receiving antioxidants
Cavallini 2004 [24]	RCT	Patients with non-operated sonographical grade IΙΙ–V varicocele and infertility receiving antioxidants × 2/day vs. placebo	2 g L-carnitine and 0.5 g acetyl-L-carnitine	6 months	62 vs. 71	No AEs or AE-related dropouts were reported in both groups
Cyrus 2015 [28]	RCT	Patients with surgically corrected clinical grade II or III varicocele and infertility receiving antioxidants × 2/day vs. placebo	250 mg vitamin C	3 months	46 vs. 69	No AEs or AE-related dropouts were reported in both groups
Ener 2016 [29]	RCT	Patients with surgically corrected clinical grade III varicocele and infertility receiving antioxidants × 2/day vs. no treatment	300 mg vitamin E	12 months	22 vs. 23	No AEs or AE-related dropouts were reported in both groups
Galatioto 2008 [30]	RCT	Patients with embolization of sonographical grade III–V varicocele and infertility receiving antioxidants × 1/day vs. no treatment	10 mg/kg N-acetyl-cysteine, 3 mg/kg vitamin C, 0.2 mg/kg vitamin E, 0.06 IU/kg vitamin A, 0.4 mg/kg thiamine, 0.1 mg/kg riboxavin, 0.2 mg/kg piridoxin, 1 mg/kg nicotinamide, 0.2 mg/kg pantothenate, 0.04 mg/kg biotin, 0.1 mg/kg cyanocobalamin, 8IU/kg ergocalciferol, 1 mg/kg calcium, 0.35 mg/kg magnesium, 0.45 mg/kg phosphate, 0.2 mg/kg iron, 0.01 mg/kg manganese, 0.02 mg/kg copper, and 0.01 mg/kg zinc	3 months	20 vs. 22	No AEs or AE-related dropouts were reported in both groups
Gamidov 2017 [31]	RCT	Patients with surgically corrected clinical grade II or III varicocele and infertility receiving antioxidants × 1/day vs. no treatment	1 g acetyl-L-carnitine, 2 g L-carnitine, 100 mg alpha-lipoic acid, 100 mg vitamin D and 4 g fructose	3 months	38 vs. 38	No AEs or AE-related dropouts were reported in both groups
Kizilay 2019 [32]	RCT	Patients with surgically corrected clinical grade I–III varicocele and infertility receiving antioxidants × 2/day vs. no treatment	1 g L-carnitine, 0.5 g acetyl-L carnitine, 1 g fructose, 50 mg citric acid, 90 mg vitamin C, 10mg zinc, 200 μg folic acid, 50 μg selenium, 20 mg CoQ10, and 1.5 μg vitamin B12	6 months	62 vs. 28	Nausea in 5 patients and gastroesophageal reflux in 4 patients receiving antioxidants
Neimark 2018 [33]	RCT	Patients with surgically corrected clinical grade III varicocele and infertility receiving antioxidants × 4/day vs. no treatment	180 mg L-arginine, 60 mg L-carnitine, 23 mg L-carnosine, 2.5 mg CoQ10, 1.5 mg glycyrrhizic acid, 1.2 mg zinc, 0.8 mg vitamin E, 0.09 mg vitamin A, and 8.5 μg selenium	6 months	38 vs. 42	No AEs or AE-related dropouts were reported in both groups
Pourmand 2014 [34]	RCT	Patients with surgically corrected clinical grade I–III varicocele and infertility receiving antioxidants × 3/day vs. no treatment	250 mg L-carnitine	6 months	50 vs. 50	Gastrointestinal reflux in patients receiving antioxidants
Povelitsa 2020 [35]	RCT	Patients with surgically corrected clinical grade III varicocele and infertility receiving antioxidants × 1/day vs. no treatment	750 mg L-carnitine, 21 mg zinc, 30 mg vitamin E, 400 μg vitamin B9, and 70 μg selenium	3 months	20 vs. 20	No AEs or AE-related dropouts were reported in both groups
Sofimajidpour 2016 [25]	Non-RCT	Patients with clinical grade II or III varicocele and infertility receiving antioxidants × 4/day vs. undergoing surgery	250 mg L-carnitine	6 months	31 vs. 31	No AEs or AE-related dropouts were reported in both groups
Zadeh 2019 [36]	RCT	Patients with surgically corrected clinical grade I–III varicocele and infertility receiving antioxidants × 1/day vs. no treatment	5 mg folic acid, 200 mg selenium, and 400 IU vitamin E	6 months	30 vs. 30	No AEs or AE-related dropouts were reported in both groups

AE: adverse event; RCT: randomized controlled study.

## Supplementary Materials

The following are available online at https://www.mdpi.com/article/10.3390/antiox10071067/s1, Data Supplement 1: PubMed search syntax and search string; Data Supplement 2: Reference list of all excluded studies with reasons for exclusion; Data Supplement 3: Risk of bias of RCTs; Data Supplement 4: Risk of bias in non-RCTs; Data Supplement 5: Forest plot of total sperm motility in patients with surgical correction of varicocele; Data Supplement 6: Forest plot of progressive sperm motility in patients with surgical correction of varicocele; Data Supplement 7: Forest plot of DNA fragmentation in patients with surgical correction of varicocele; Data Supplement 8: Grading of evidence for all outcomes.
