# Supplementary material for: The Effect of Antioxidant Supplementation on Operated or Non-Operated Varicocele-Associated Infertility: A Systematic Review and Meta-Analysis
Source: Antioxidants (Basel). 2021 Jul 1;10(7):1067. doi: 10.3390/antiox10071067 (PMC8301171; doi:10.3390/antiox10071067)
Supplement: Supplementary file 1 [file antioxidants-10-01067-s001.zip › antioxidants-1265304-supplementary.pdf]

Data Supplement

Table of Contents

Data Supplement 1: PubMed search syntax and search string **..** ***2***

Data Supplement 2: Reference list of all excluded studies with reasons for exclusion **........** ***4***

Data Supplement 3: Risk of bias of RCTs **..** ***6***

Data Supplement 4: Risk of bias in non-RCTs **..** ***7***

Data Supplement 5: Forest plot of total sperm motility in patients with surgical correction of varicocele **..** ***8***

Data Supplement 6: Forest plot of progressive sperm motility in patients with surgical correction of varicocele **...** ***9***

Data Supplement 7: Forest plot of DNA fragmentation in patients with surgical correction of varicocele **..** ***10***

Data Supplement 8: Grading of evidence for all outcomes **..** ***11***

Data Supplement 1: PubMed search syntax and search string

Search syntax

ID	Search
#1	varicocele [All Fields]
#2	varicocele [MeSH terms]
#3	varicocelectomy [All Fields]
#4	OR #1-3
#5	antioxidant [All Fields]
#6	antioxidants [MeSH terms]
#7	vitamin [All Fields]
#8	selenium [All Fields]
#9	folic acid [All Fields]
#10	glutathione [All Fields]
#11	ascorbic acid [All Fields]
#12	zinc [All Fields]
#13	fatty acids [All Fields]
#14	carnitine [All Fields]
#15	oil [All Fields]
#16	pentoxifylline [All Fields]
#17	ubiquinol [All Fields]
#18	omega [All Fields]
#19	folate [All Fields]
#20	OR #5-19
#21	#4 AND #20

Search string

("varicocele"[MeSH Terms] OR "varicocele"[All Fields] OR "varicoceles"[All Fields] OR "varicocelized"[All Fields] OR "varicocoele"[All Fields] OR "varicocoeles"[All Fields] OR ("varicocelectomies"[All Fields] OR "varicocelectomy"[All Fields])) AND ("antioxidant s"[All Fields] OR "antioxidants"[Pharmacological Action] OR "antioxidants"[MeSH Terms]

OR "antioxidants"[All Fields] OR "antioxidant"[All Fields] OR "antioxidating"[All Fields] OR "antioxidation"[All Fields] OR "antioxidative"[All Fields] OR "antioxidatively"[All Fields] OR "antioxidatives"[All Fields] OR "antioxidizing"[All Fields] OR ("vitamin s"[All Fields] OR "vitamine"[All Fields] OR "vitamines"[All Fields] OR "vitamins"[Pharmacological Action] OR "vitamins"[MeSH Terms] OR "vitamins"[All Fields] OR "vitamin"[All Fields]) OR "selenium"[All Fields] OR "folic acid"[All Fields] OR "glutathione"[All Fields] OR "ascorbic acid"[All Fields] OR "zinc"[All Fields] OR "fatty acids"[All Fields] OR ("carnitin"[All Fields] OR "carnitine"[MeSH Terms] OR "carnitine"[All Fields] OR "carnitine s"[All Fields] OR "carnitines"[All Fields]) OR "oil"[All Fields] OR ("pentoxifylline"[MeSH Terms] OR "pentoxifylline"[All Fields] OR "pentoxifyllin"[All Fields]) OR ("ubiquinol"[Supplementary Concept] OR "ubiquinol"[All Fields] OR "ubiquinols"[All Fields]) OR ("omega"[All Fields] OR "omegae"[All Fields] OR "omegas"[All Fields]) OR ("folic acid"[MeSH Terms] OR ("folic"[All Fields] AND "acid"[All Fields]) OR "folic acid"[All Fields] OR "folate"[All Fields] OR "folates"[All Fields]))

<u>The search strategy was modified accordingly for the other databases.</u>

Data Supplement 2: Reference list of all excluded studies with reasons for exclusion

<u>Duplicate publications or data presented in part and then in whole in future publications</u>

1. Azizollahi G, Azizollahi S, Babaei H, Kianinejad MA, Baneshi MR, Nematollahi-Mahani SN. Effects of zinc sulfate and folic acid coadministration on sperm parameters, protamine content and acrosomal integrity of varicocelectomized patients. Iranian journal of reproductive medicine. 2013;11:37--37-.
2. Busetto GM, Agarwal A, Virmani A, Del Giudice F, Micic S, Gentile V, et al. Pregnancy rate and infertility in patients with varicocele and/or oligoasthenoteratozoospermia: evaluation of antioxidant supplementation effect on sperm parameters. HUMAN REPRODUCTION. 2018;33(1):153–4.
3. Busetto GM, Del Giudice F, Virmani A, Sciarra A, Maggi M, Ferro M, et al. Body mass index and age correlate with antioxidant supplementation effects on sperm quality: Post hoc analyses from a double-blind placebo-controlled trial. Andrologia [Internet]. 2020;52(3). Available from: https://www.scopus.com/inward/record.uri?eid=2-s2.0-85078889721&doi=10.1111%2Fand.13523&partnerID=40&md5=ed2fb47a09ce72a4e2b10577bcb7d242
4. Busetto GM, Virmani A, Antonini G, Ragonesi G, Del Giudice F, Gentile V, et al. Effect of antioxidant supplementation on sperm parameters in oligoastheno-teratozoospermia, with and without varicocele: a double blind place controlled (DBPC) study. European urology, supplements. 2017;16(1):142–3.
5. Busetto GM, Virmani A, Del Giudice F, Micic S, Agarwal A, De Berardinis E, et al. Varicocele and oligoasthenoteratozoospermia: evaluation of antioxidant supplementation effect on pregnancy rate and sperm quality. European urology, supplements. 2017;108(3):e133--e133-.
6. Busetto GM, Virmani MA, Antonini G, Ragonesi G, De Berardinis E, Agarwal A, et al. Effect of antioxidant supplementation on sperm parameters in oligoasthenoteratozoospermia, with and without varicocele: a DBPC study. Fertility and sterility. 2016;106(3):e46–e46.
7. Busetto G, Virmani MA, Del Giudice F, Micic S, Agarwal A, De Berardinis E. Body mass index & age correlate with antioxidants supplementation effect on sperm quality: a double-blind placebo controlled trial on patients with varicocele & oligoasthenoteratozoospermia. Fertility and Sterility. 2018;110(4):E158–E158.
8. Busetto GM, Del Giudice F, Agarwal A, Micic S, Virmani A, De Berardinis E. Body mass index and age correlate with antioxidants supplementation effect on sperm quality: a double blind place controlled trial on patients with varicocele & oligoasthenoteratozoospermia. Journal of Urology. 2019;201(4):E769–E769.
9. Busetto GM, Del Giudice F, Virmani A, Sciarra A, Maggi M, Ferro M, et al. Body mass index and age correlate with antioxidant supplementation effects on sperm quality: Post hoc analyses from a double-blind placebo-controlled trial. Andrologia. 2020;52(3):e13523–e13523.
10. Nematollahi-Mahani SN, Azizollahi GH, Baneshi MR, Safari Z, Azizollahi S. Effect of folic acid and zinc sulphate on endocrine parameters and seminal antioxidant level after varicocelectomy. Andrologia. 2014;46(3):240–5.
11. Ovchinnikov R, Gamidov S, Popova A. Efficacy of adjuvant antioxidant therapy after microsurgical varicocelectomy in infertile men. Andrology. 2018;6:63--63-.
12. Pourmand GH, Movahedin M, Dehghan S, Mehrsai A, Ahmadi A, Pourhosein M. Does L-carnitine therapy add any extra benefit to standard inguinal varicocelectomy in terms of deoxyribonucleic acid damage or sperm quality factor indices: a randomized study. Iranian journal of reproductive medicine. 2015;13(4):67–67.
13. Pourmand G, Movahedin M, Noori M, Dehghani S, Hoseini M, Ziloochi M. Does anti-oxidant therapy add any extra benefit to standard inguinal varicocelectomy in terms of DNA damage or sperm quality factor indices: a randomized study. International journal of fertility and sterility. 2014;8:53–53.
14. Pourmand G, Movahedin M, Noori M, Dehghani S, Hosseini M, Ziloochi M, et al. Does antioxidant therapy add any benefit in improvement of dna damage to standard inguinal varicocelectomy? A randomized case-control study. Journal of Urology. 2014;191(4):E732–E732.

<u>Ineligible intervention</u>

1. Asr Badr YA, Sepehran E, Del Azar A, Sadeghi H, Nouri M. The Effect of Saffron on Semen Analysis in Infertile Men with Clinical Varicocele After Varicocelectomy. Nephro-Urol Mon. 2017;9(5):e59939.
2. Azadi L, Abbasi H, Deemeh MR, Tavalaee M, Arbabian M, Pilevarian AA, et al. Zaditen (Ketotifen), as mast cell blocker, improves sperm quality, chromatin integrity and pregnancy rate after varicocelectomy. Int J Androl. 2011;34(5):446–52.

3. Cavallini G, Biagiotti G, Ferraretti AP, Gianaroli L, Vitali G. Medical therapy of oligoasthenospermia associated with left varicocele. BJU Int. 2003;91(6):513–8.
4. Deemeh M, Arbabian M, Tavalaee M, Abbasi H, Nasr- Esfahani MH. Comparison of Zaditen and NAC on semen parameters of individuals with varicocele after varicocelectomy. Iranian journal of reproductive medicine. 2014;12(6):39–39.
5. Fang Y, Zhao L, Yan F, Xia X, Xu D, Cui X. Escin improves sperm quality in male patients with varicocele-associated infertility. Phytomedicine. 2010;17(3):192–6.
6. Gamidov SI, Ovchinnikov RI, Popova AVu, Tkhagapsoveva RA, Izhbayev SKh. Current approach to therapy for male infertility in patients with varicocele. Terapevticheskii Arkhiv. 2012;84(10):56–61.
7. Lozano-Hernandez R, Velasco J, Juarez A, Machado JE. Evaluation of seminal parameters in varicocelectomized men treated with lepidium meyenni (maca). Acta Bioclinica. 2019;9(17):91–102.
8. Lu X-LX-L, Liu J-JJ-J, Li J-TJ-T, Yang Q-AQ-A, Zhang J-MJ-M. Melatonin therapy adds extra benefit to varicecelectomy in terms of sperm parameters, hormonal profile and total antioxidant capacity: A placebo-controlled, double-blind trial. Andrologia. 2018;50(6):e13033–e13033.
9. Mićić S, Tulić C, Dotlić R. Kallikrein therapy of infertile men with varicocele and impaired sperm motility. Andrologia. 1990;22(2):179–83.
10. Pan Z, Deng N, Zou Z. Clinical observation of effect of diosmin combined with Jinshuibao capsule treatment on improvement of semen quality in patients with varicocele. Medical Science Technology. 2016;57:42–6.
11. Park HJ, Choe S, Park NC. Effects of Korean red ginseng on semen parameters in male infertility patients: A randomized, placebo-controlled, double-blind clinical study. Chin J Integr Med. 2016;22(7):490–5.
12. Qu X, Shan Z, Zhang N, Guo L. Curative effect of surgery in combination with compound xuanju capsule in treating subclinical varicocele induced infertility. Biomedical Research-tokyo. 2017;28:1247–50.
13. Söylemez H, Kiliç S, Atar M, Penbegül N, Sancaktutar AA, Bozkurt Y. Effects of micronised purified flavonoid fraction on pain, semen analysis and scrotal color Doppler parameters in patients with painful varicocele; results of a randomized placebo-controlled study. Int Urol Nephrol. 2012;44(2):401–8.
14. Takahara H, Cosentino MJ, Cockett ATK. Zinc therapy along or in combination with varicocelectomy to improve the fertility potential of the male. Journal of andrology. 1982;3(1):37–37.
15. Takihara H, Cosentino MJ, Cockett AT. Zinc sulfate therapy for infertile male with or without varicocelectomy. Urology. 1987;29(6):638–41.
16. Yingjun W, Weili Z, Dawen L. Effects of Shi Zi Er Xian decoction on sperm acrosomal integrity and DNA integrity in patients after varicocelectomy. Chinese journal of andrology. 2012;26(3):19–23.
17. Zaazaa A, Adel A, Fahmy I, Elkhiat Y, Awaad AA, Mostafa T. Effect of varicocelectomy and/or mast cells stabilizer on sperm DNA fragmentation in infertile patients with varicocele. Andrology. 2018;6(1):146–50.
18. Zhukov OB, Bragina EE, Levina AV, Evdokimov VV, Terushkin RA, Akramov MM, et al. Comparison of the effectiveness of medications containing a combination of zinc and arginine for treatment of male infertility. Andrologia i Genital'naa Hirurgia. 2020;21(2):26–35.

Single-arm study design

1. Festa R, Giacchi E, Raimondo S, Tiano L, Zuccarelli P, Silvestrini A, et al. Coenzyme Q10 supplementation in infertile men with low-grade varicocele: An open, uncontrolled pilot study. Andrologia. 2014;46(7):805–7.
2. Gual-Frau J, Abad C, Amengual MJ, Hannaoui N, Checa MA, Ribas-Maynou J, et al. Oral antioxidant treatment partly improves integrity of human sperm DNA in infertile grade i varicocele patients. Human Fertility. 2015;18(3):225–9.
3. Ianniello B, Gambardella V, Giannotti F, D'Antò V, Vitelli A, D'Ettore A, et al. Support therapy with integrators after varicocelectomy. Giornale Italiano di Ostetricia e Ginecologia. 2004;26(12):469–72.

Data Supplement 3: Risk of bias of RCTs

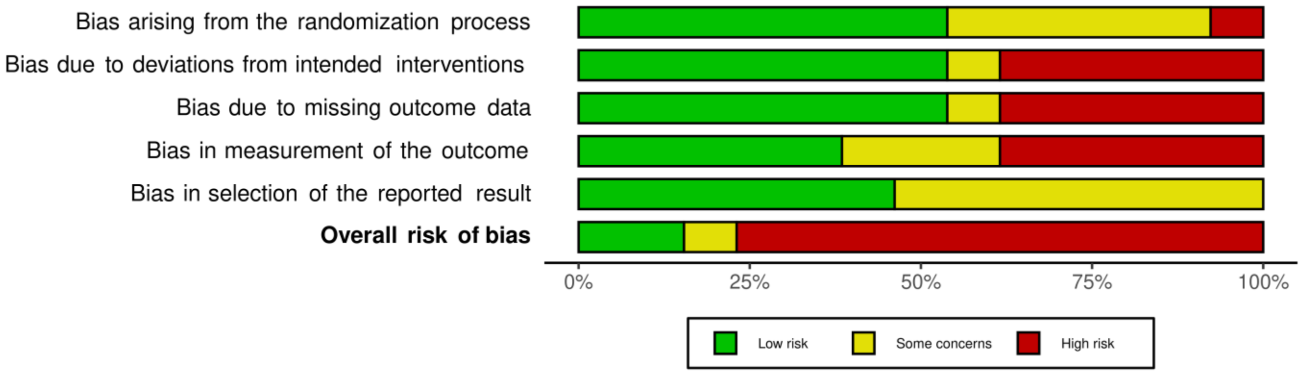

Data Supplement 3.1: Overall risk of bias assessment according to the RoB 2 tool for RCTs.

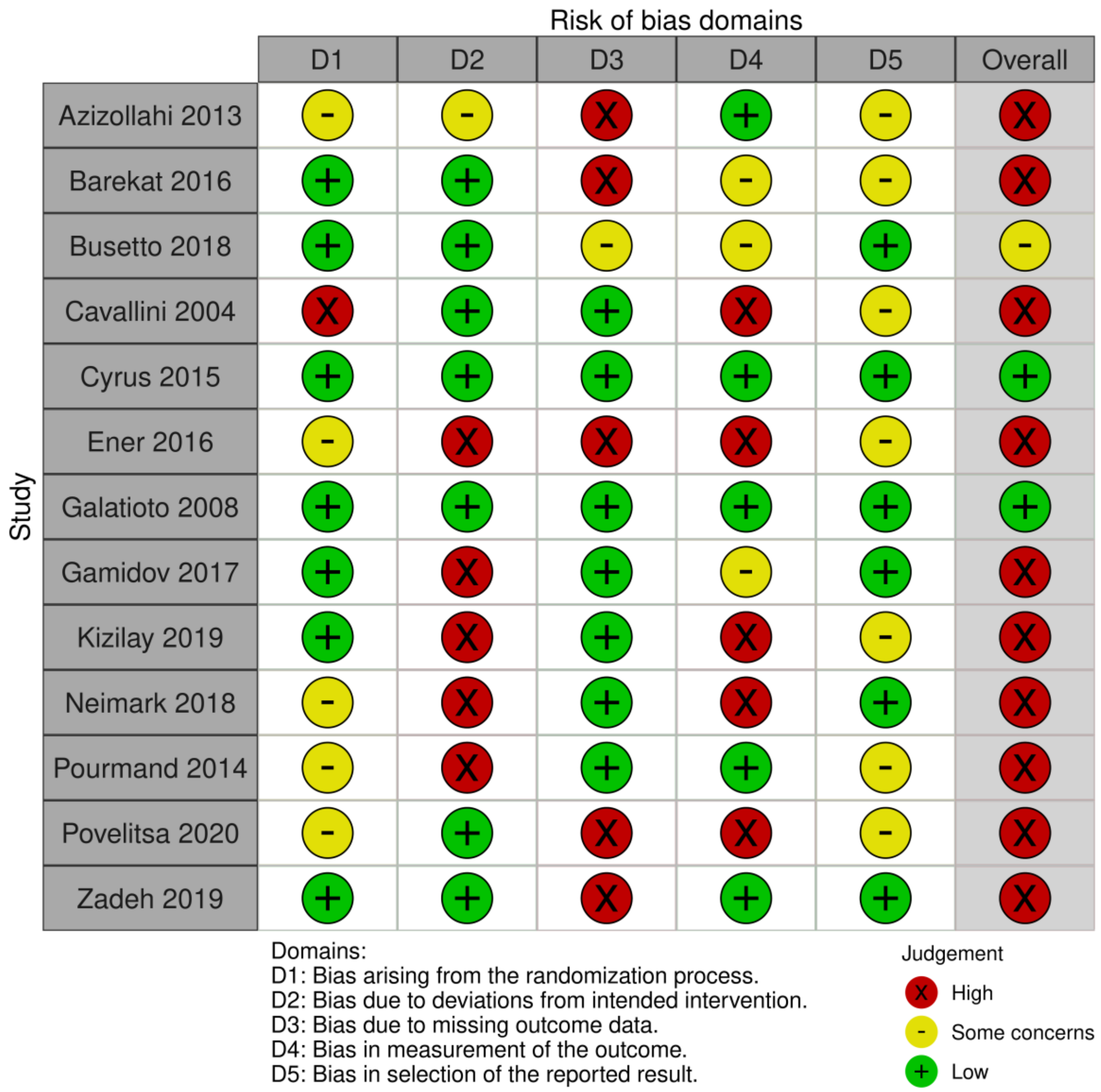

Data Supplement 3.2: Study-by-study risk of bias assessment according to the RoB 2 tool for RCTs.

Data Supplement 4: Risk of bias in non-RCTs

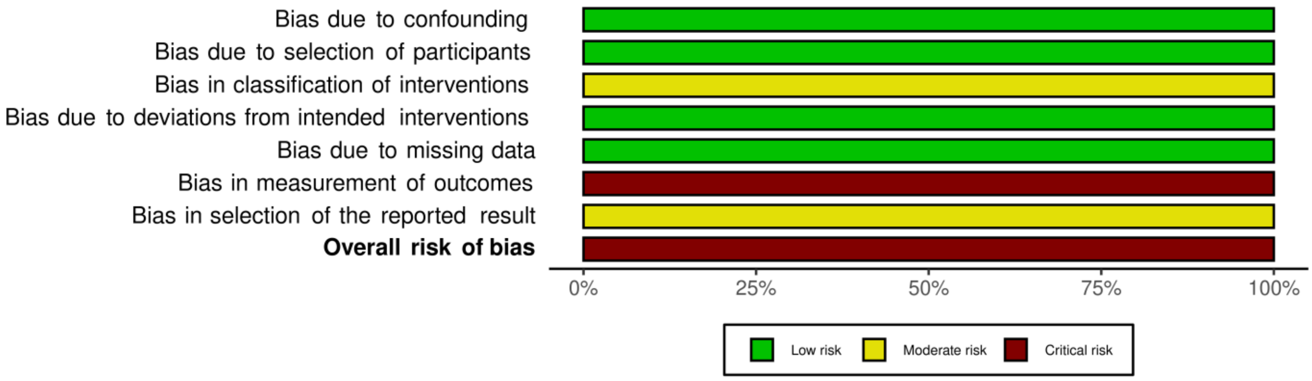

Data Supplement 4.1: Overall risk of bias assessment in included non-RCTs.

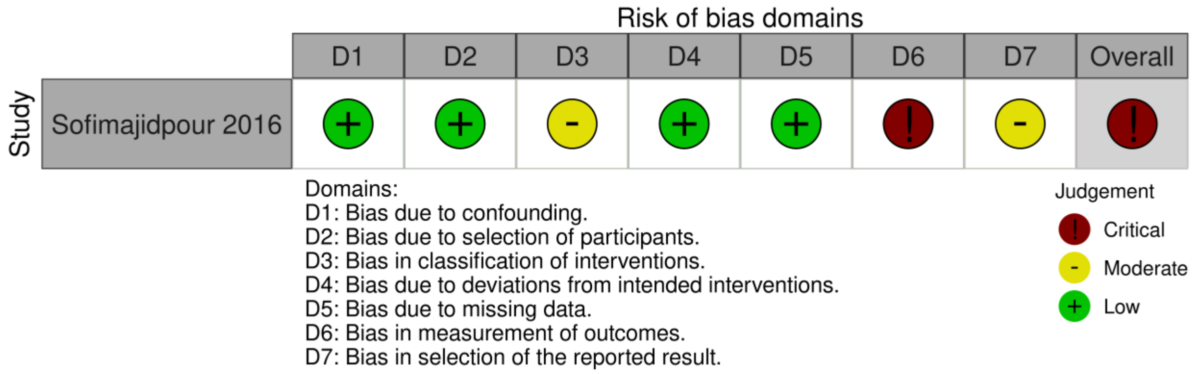

Data Supplement 4.2: Study-by-study risk of bias assessment in included non-RCTs.

Data Supplement 5: Forest plot of total sperm motility in patients with surgical correction of varicocele

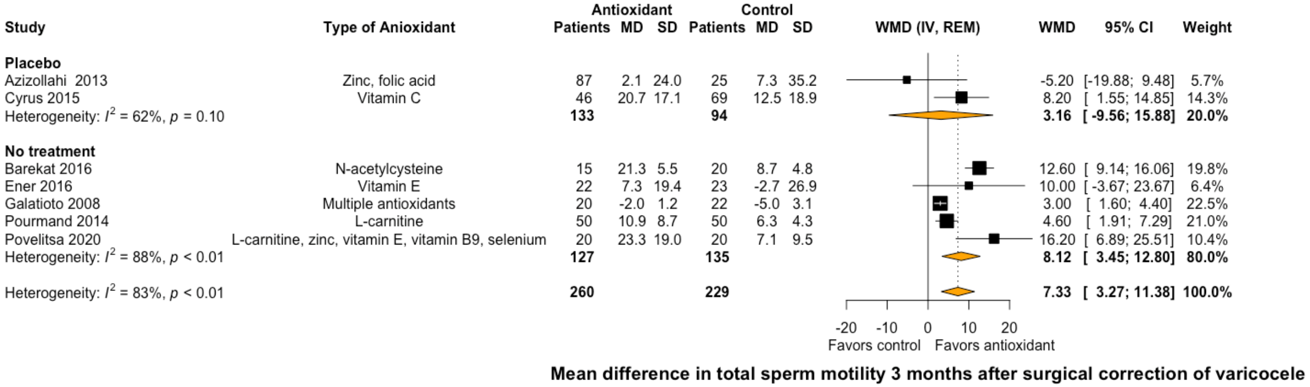

Data Supplement 5.1: Forest plot of mean difference in total sperm motility at 3 months after treatment with antioxidants versus placebo or no treatment in patients with surgical correction of varicocele. CI: confidence interval; IV: inverse variance; MD: mean difference; REM: random effects model; SD: standard deviation; WMD: weighted mean difference.

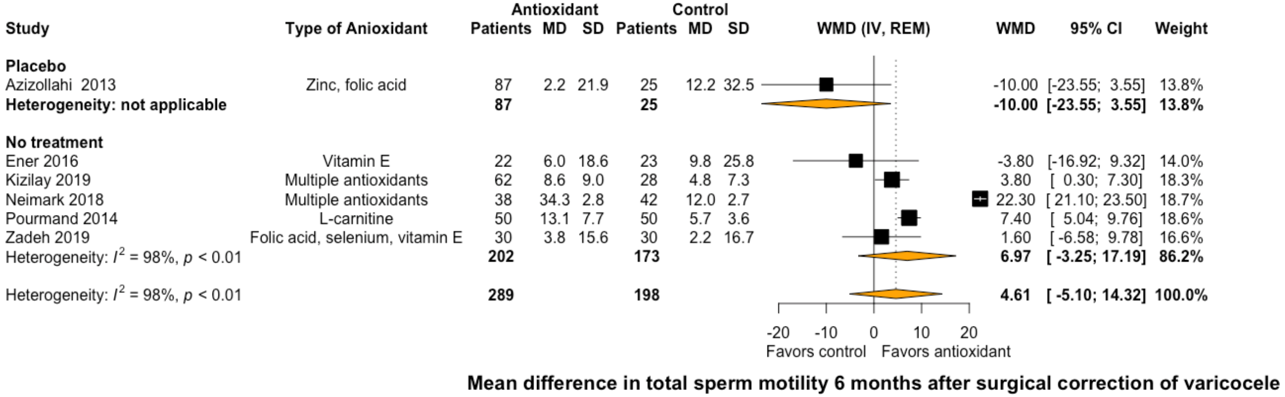

Data Supplement 5.2: Forest plot of mean difference in total sperm motility at 6 months after treatment with antioxidants versus placebo or no treatment in patients with surgical correction of varicocele. CI: confidence interval; IV: inverse variance; MD: mean difference; REM: random effects model; SD: standard deviation; WMD: weighted mean difference.

Data Supplement 6: Forest plot of progressive sperm motility in patients with surgical correction of varicocele

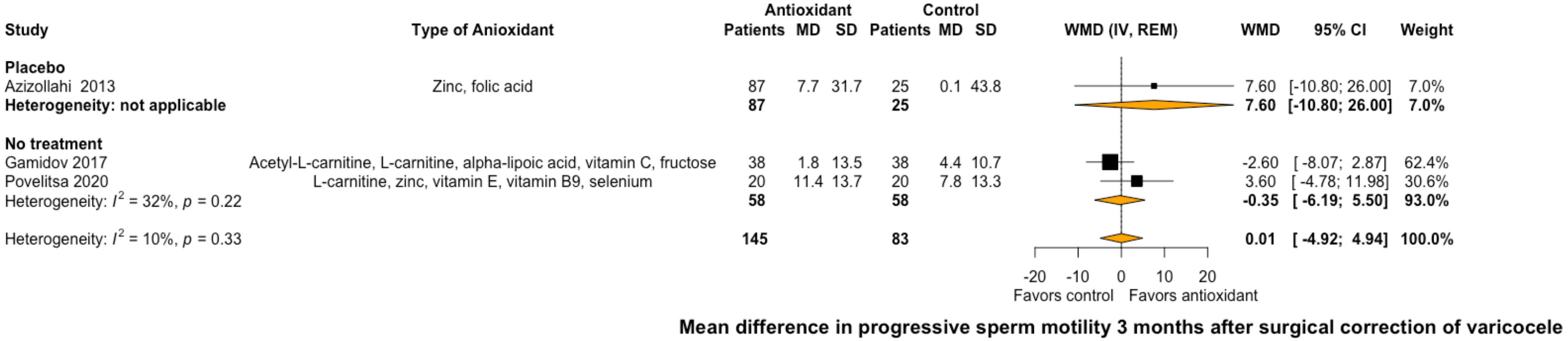

Data Supplement 6.1: Forest plot of mean difference in progressive sperm motility at 3 months after treatment with antioxidants versus placebo or no treatment in patients with surgical correction of varicocele. CI: confidence interval; IV: inverse variance; MD: mean difference; REM: random effects model; SD: standard deviation; WMD: weighted mean difference.

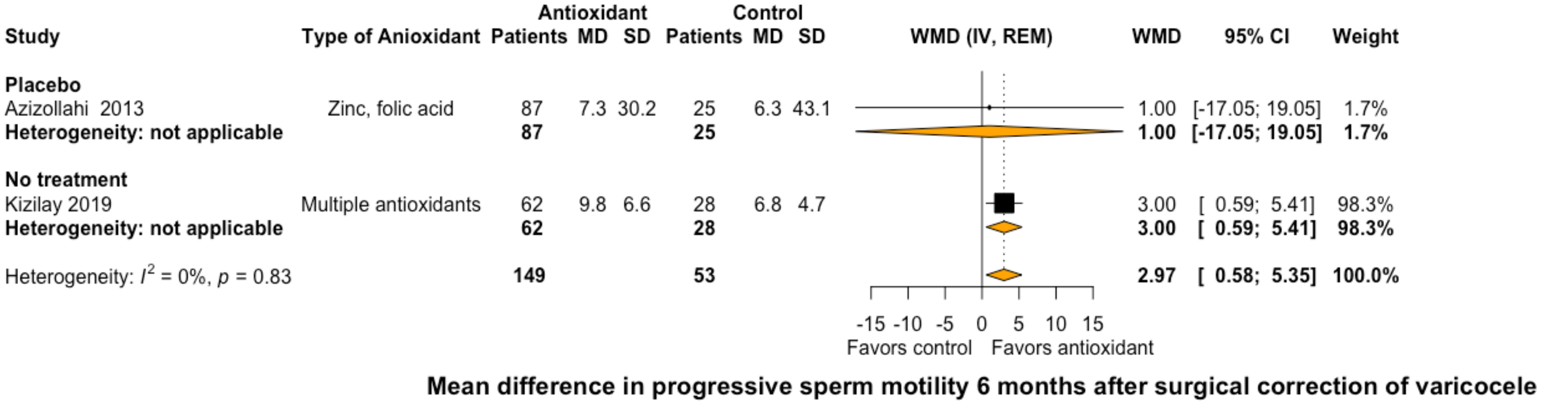

Data Supplement 6.2: Forest plot of mean difference in progressive sperm motility at 6 months after treatment with antioxidants versus placebo or no treatment in patients with surgical correction of varicocele. CI: confidence interval; IV: inverse variance; MD: mean difference; REM: random effects model; SD: standard deviation; WMD: weighted mean difference.

Data Supplement 7: Forest plot of DNA fragmentation in patients with surgical correction of varicocele

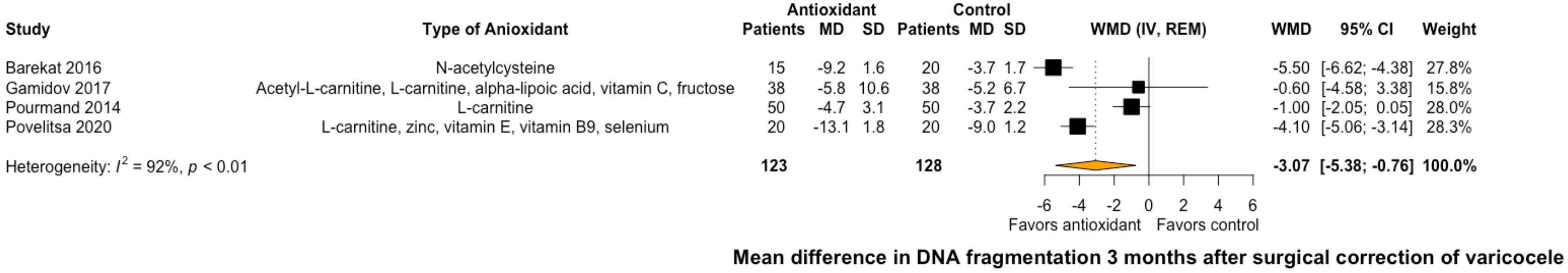

Data Supplement 7.1: Forest plot of mean difference in DNA fragmentation at 3 months after treatment with antioxidants versus placebo or no treatment in patients with surgical correction of varicocele. CI: confidence interval; IV: inverse variance; MD: mean difference; REM: random effects model; SD: standard deviation; WMD: weighted mean difference.

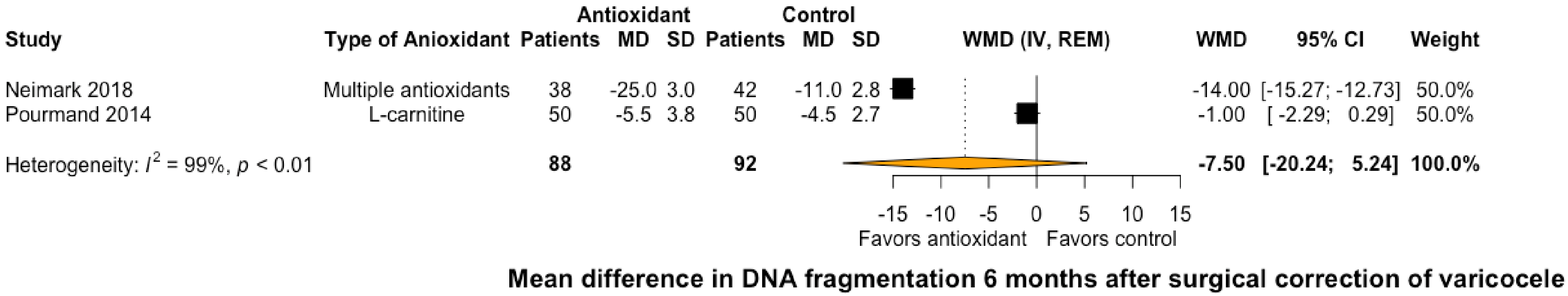

Data Supplement 7.2: Forest plot of mean difference in DNA fragmentation at 6 months after treatment with antioxidants versus placebo or no treatment in patients with surgical correction of varicocele. CI: confidence interval; IV: inverse variance; MD: mean difference; REM: random effects model; SD: standard deviation; WMD: weighted mean difference.

Data Supplement 8: Grading of evidence for all outcomes

Certainty assessment							№ of patients		Effect		Certainty	Importance
**№ of studies**	**Study design**	**Risk of bias**	**Inconsistency**	**Indirectness**	**Imprecision**	**Other considerations**	**Antioxidant**	**Control**	**Relative (95% CI)**	**Absolute (95% CI)**		
Pregnancy rate at 3 months												
2	randomized trials	serious [a]	not serious	not serious	serious [b]	none	9/53 (17%)	5/58 (8.6%)	**OR 2.28** (0.7 to 7.48)	**91 more per 1000** (from 24 fewer to 328 more)	⊕⊕◯◯ LOW	Important
Pregnancy rate at 6 months												
1	randomized trials	serious [a]	not serious	not serious	serious [b]	none	18/62 (29%)	5/28 (17.9%)	**OR 2.58** (1.03 to 6.45)	**112 more per 1000** (from 60 fewer to 376 more)	⊕⊕◯◯ LOW	Important
Sperm concentration at 3 months												
7	randomized trials	serious [a]	not serious	not serious	serious [b]	none	248	217	Not applicable	MD **9.25 higher** (6.41 higher to 12.09 higher)	⊕⊕◯◯ LOW	Important
Sperm concentration at 6 months												
5	randomized trials	serious [a]	serious [c]	not serious	serious [b]	none	239	148	Not applicable	MD **5.92 higher** (6.76 lower to 18.6 higher)	⊕◯◯◯ VERY LOW	Important
Normal sperm morphology at 3 months												

Certainty assessment							№ of patients		Effect		Certainty	Importance
№ of studies	Study design	Risk of bias	Inconsistency	Indirectness	Imprecision	Other considerations	Antioxidant	Control	Relative (95% CI)	Absolute (95% CI)		
7	randomized trials	serious [a]	serious [c]	not serious	serious [b]	none	276	244	Not applicable	MD **1.86 higher** (0.85 higher to 2.86 higher)	⊕◯◯◯ VERY LOW	Important
Normal sperm morphology at 6 months												
5	randomized trials	serious [a]	serious [c]	not serious	serious [b]	none	267	175	Not applicable	MD **5.19 higher** (1.88 lower to 12.63 higher)	⊕◯◯◯ VERY LOW	Important
Total sperm motility at 3 months												
7	randomized trials	serious [a]	serious [c]	not serious	serious [b]	none	260	229	Not applicable	MD **7.33 higher** (3.27 higher to 11.38 higher)	⊕◯◯◯ VERY LOW	Important
Total sperm motility at 6 months												
6	randomized trials	serious [a]	serious [c]	not serious	serious [b]	none	289	198	Not applicable	MD **4.61 higher** (5.1 lower to 14.32 higher)	⊕◯◯◯ VERY LOW	Important
Progressive sperm motility at 3 months												
3	randomized trials	serious [a]	not serious	not serious	serious [b]	none	145	83	Not applicable	MD **0.01 higher** (4.92 lower to 4.94 higher)	⊕⊕◯◯ LOW	Important

Progressive sperm motility at 6 months

Certainty assessment							№ of patients		Effect		Certainty	Importance
**№ of studies**	**Study design**	**Risk of bias**	**Inconsistency**	**Indirectness**	**Imprecision**	**Other considerations**	**Antioxidant**	**Control**	**Relative (95% CI)**	**Absolute (95% CI)**		
2	randomized trials	serious [a]	not serious	not serious	serious [b]	none	149	53	Not applicable	MD **2.97 higher** (0.58 higher to 5.35 higher)	⊕⊕◯◯ LOW	Important
**DNA fragmentation at 3 months**												
4	randomized trials	serious [a]	serious [c]	not serious	serious [b]	none	123	128	Not applicable	MD **3.07 lower** (5.38 lower to 0.76 lower)	⊕◯◯◯ VERY LOW	Important
**DNA fragmentation at 6 months**												
2	randomized trials	serious [a]	serious [c]	not serious	serious [b]	none	88	92	Not applicable	MD **7.5 lower** (20.24 lower to 5.24 higher)	⊕◯◯◯ VERY LOW	Important

CI: confidence interval; MD: mean difference; OR: odds ratio. [a]most included studies at high or unclear risk of bias; [b]small sample size; [c]statistically significant heterogeneity among studies.
